# Age-Dependent Transition from Cell-Level to Population-Level Control in Murine Intestinal Homeostasis Revealed by Coalescence Analysis

**DOI:** 10.1371/journal.pgen.1003326

**Published:** 2013-02-28

**Authors:** Zheng Hu, Yun-Xin Fu, Anthony J. Greenberg, Chung-I Wu, Weiwei Zhai

**Affiliations:** 1 Center for Computational Biology and Laboratory of Disease Genomics and Individualized Medicine, Beijing Institute of Genomics, Chinese Academy of Sciences, Beijing, China; 2 Graduate University of Chinese Academy of Sciences, Beijing, China; 3 Human Genetics Center and Division of Biostatistics, School of Public Health, University of Texas Health Science Center at Houston, Houston, Texas, United States of America; 4 Departments of Biological Statistics and Computational Biology, Cornell University, Ithaca, New York, United States of America; 5 Department of Ecology and Evolution, University of Chicago, Chicago, Illinois, United States of America; 6 National Center for Mathematics and Interdisciplinary Sciences, Chinese Academy of Sciences, Beijing, China; New York University, United States of America

## Abstract

In multi-cellular organisms, tissue homeostasis is maintained by an exquisite balance between stem cell proliferation and differentiation. This equilibrium can be achieved either at the single cell level (a.k.a. cell asymmetry), where stem cells follow strict asymmetric divisions, or the population level (a.k.a. population asymmetry), where gains and losses in individual stem cell lineages are randomly distributed, but the net effect is homeostasis. In the mature mouse intestinal crypt, previous evidence has revealed a pattern of population asymmetry through predominantly symmetric divisions of stem cells. In this work, using population genetic theory together with previously published crypt single-cell data obtained at different mouse life stages, we reveal a strikingly dynamic pattern of stem cell homeostatic control. We find that single-cell asymmetric divisions are gradually replaced by stochastic population-level asymmetry as the mouse matures to adulthood. This lifelong process has important developmental and evolutionary implications in understanding how adult tissues maintain their homeostasis integrating the trade-off between intrinsic and extrinsic regulations.

## Author Summary

In multi-cellular organisms, there is a static equilibrium maintaining cells of various forms. This homeostasis is achieved by an exquisite balance between stem cell proliferation and differentiation. Understanding how different species and organ types maintain this dynamic equilibrium has been an interesting question for both evolutionary and developmental biologists. Using population genetic theory together with previously published single-cell sequencing data collected from mouse intestinal crypts at two points in development, we have revealed a dynamic picture of stem cell renewal in intestinal crypts. We found that intestinal equilibrium is maintained at the single-cell level through predominantly asymmetric stem cell divisions at early life stages, but progressively switches to a population level homeostasis with only symmetric divisions as the mouse matures to adulthood. This dynamic process, likely to be conserved across species, has important developmental and evolutionary implications in understanding how adult tissues maintain their homeostasis integrating lifelong trade-offs between intrinsic and extrinsic factors.

## Introduction

Development and tissue homeostasis of multi-cellular organisms is an extraordinary cellular orchestra starting from a single zygote [1]. Cascades of cell divisions generate and subsequently maintain a great diversity of cells in an organism [2]. This life-long balance is strictly controlled and maintained through a rigid cellular hierarchy, where the stem cells lie at the apex of the division cascades [3].

Stem cells are a group of cells with a dual role. On one hand, they need to maintain their own population through self-renewal. On the other hand, stem cells also give rise to differentiated cells which carry out most body functions [4]. In order to fulfill the dual role of self-renewal and differentiation, stem cells can undergo two different modes of cell division – asymmetric and symmetric [5]. In the asymmetric division mode, one daughter cell is maintained as the stem cell and the other goes on and evolves into terminally differentiated cells. The stem cells can also divide symmetrically, leading to either two stem cells or two differentiated cells.

Asymmetric division is particularly attractive and allows stem cells to accomplish both maintenance and differentiation simultaneously in a single division. However, symmetric divisions are also indispensable in situations such as morphogenesis and tissue injury where stem cells need to proliferate rapidly [6], [7]. A robust balance between proliferation and differentiation must be maintained to prevent aberrant growth on one hand and tissue loss on the other [5].

Stem cells often form distributed clusters and live in local nurtured structures known as the stem cell niches [8], [9]. In order to maintain a static hierarchy between different cell types, two different strategies can be employed. In the first strategy (also called cell asymmetry) [10], stem cells engage only in asymmetric divisions where dual roles of self renewal and differentiations can be successfully fulfilled while keeping the stem cell number constant. Population level equilibrium is achieved by maintaining a stasis at the single cell level through asymmetric cell divisions. Studies looking at invertebrate systems, in particular *Drosophila melanogaster* and *Caenorhabditis elegans,* have found a predominance of asymmetric divisions where stem daughter cells remain within the niche and differentiated cells exit and evolve into functional cells [11], [12]. Biological evidence for cell asymmetry is quite strong in many invertebrate systems [10]. In the other extreme (also called population asymmetry), each stem cell division gives rise to one stem cell and one differentiated cell on average [10]. Homeostasis is maintained by having a subset of cells proliferate while other stem cells are lost through differentiation. If the gain and loss are balanced, stasis is achieved at the population level rather than at the level of individual cell divisions [13]. In contrast to invertebrates, it appears that population asymmetry is more prevalent in mammals [14].

Mammalian intestine has become one of the best model systems for studying stem cell dynamics [15]–[18]. Powerful genetic tools together with recently-identified intestinal stem cell markers enable us to directly trace stem cells [19]–[21]. As a result, dynamics of cell populations are becoming accessible to investigation [22]. Interestingly, the relative prevalence of symmetric and asymmetric divisions among studies conducted to date is not yet clear. On one hand, lineage tracing techniques together with models from statistical physics reveal a pattern of neutral drift in a group of equipotent stem cells [23], [24]. Population asymmetry with a predominance of symmetric divisions is the major mode of stem cell renewal in the adult mouse intestinal crypt. On the other hand, optimal control theory together with experimental data indicates that early development of the mouse intestinal crypt is achieved by a surge of symmetric divisions establishing the stem population followed by a transition to predominantly asymmetric divisions [25]. Moreover, molecular evidence for asymmetric division is starting to accumulate, suggesting that the role of asymmetric divisions might be underappreciated [26]. The dynamics of stem cell renewal, in particular the balance between cell asymmetry and population asymmetry of stem cells, remain enigmatic.

Genomic sequencing, in particular single cell sequencing, provides a powerful alternative approach for studying cell lineage relationships. Compared to traditional molecular techniques such as the lineage tracing [27], spontaneous somatic mutations provide a natural internal cell marker for tracing relationships in a group of cells. In this work, we use a previously published dataset comprising single-cell sequence information collected from mouse intestinal crypts [28]. The authors of this study sequenced multiple microsatellite markers in a repair-deficient mouse strain (Mlh1−/−) [28], [29]. The reduced efficiency of DNA repair machinery results in higher microsatellite mutation rate and thus increased genetic variation, allowing us to discern genealogical relationships in this group of cells. Using traditional phylogenetic methods, the authors of the previous study found that intestinal crypts do not support the immortal strand hypothesis. Instead, they found support for the existence of monoclonal conversion, a process by which multiple crypt cells drift toward monoclonality, where offspring population is only derived from a single ancestor [28].

Population-genetic theory [30], in particular coalescent theory [31], [32], provides a natural framework for studying cells in a population. Population dynamics driven by a combination of symmetric and asymmetric divisions can be explicitly modeled. When we take a sample of cells from a tissue, there will be a genealogical relationship relating individual cells to their common ancestor [33]. The shape of this genealogy is governed by the mode of cell division and thus carries information about underlying population dynamics. In reality, we do not directly observe the genealogy, but rather genotype information (e.g. microsatellite markers presented here) collected from individual cells. By considering all possible ancestral relationships compatible with a given pattern of genetic variation (instead of just a single gene genealogy in the phylogenetic framework [28]), we can infer the underlying balance between symmetric and asymmetric divisions using statistical modeling. Here, we demonstrate that this approach, based on classical population genetics, can provide powerful insights into cellular dynamics within an organism and supply fine-scale quantitative description of the processes underlying cellular homeostasis.

## Results

### A population-genetic model with stem cell division mode and coalescent

**Figure 1:**
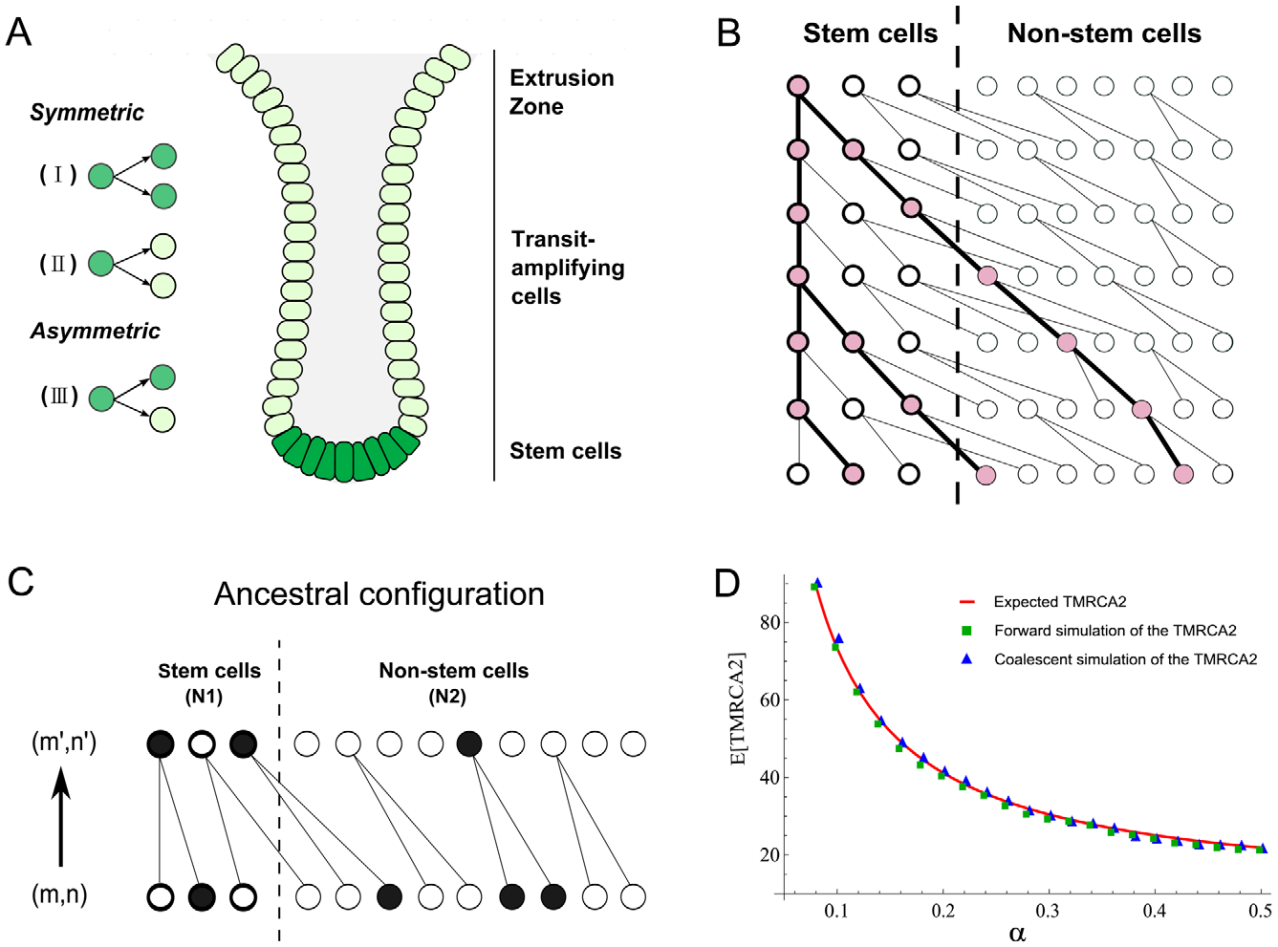
Anatomy of intestinal crypts and coalescent processes for the two-deme model. (A) Anatomical structure of the intestinal crypt. The dark green cells represent stem cells and light green cells are transit-amplifying cells. There are three types of stem cells divisions (I, II and III, see main text). (B) A cartoon illustration of a coalescent process in the two deme model. One cell from stem cell deme and two cells from the transit-amplifying cell deme were sampled. Their ancestral relationship is depicted as the gene tree connecting their ancestors. (C) The state transition for the Markov Chain in one step. The current state of the chain is (m,n) and the state in the previous generation is (m′,n′). In the example here, (m,n) = (1,3) and (m′,n′) = (2,1). (D) The expected time to the most recent common ancestor for two lineages (denoted as TMRCA2) was calculated using three different approaches. The solid line is calculated using a first-step analysis of the Markov Chain. Blue squares are the results from the forward simulation and triangles are from the direct simulation following the Markov Chain.

We consider a discrete-generation model of tissue homeostasis. In each cell generation, a proportion α of the cells divides symmetrically and gives rise to two descendant stem cells (type I, Figure 1A). A fraction β of the cells divides asymmetrically (type III) and 1-α-β cells divide symmetrically and produce two differentiated cells (type II). Because type II divisions do not give rise to any stem cell descendants, the number of stem cells in generation t will be N_t_ = (2α+β)^t^×N_0_, where N_0_ is the population size at time 0.

Now, suppose we pick two stem cells at random at time t, the probability that they will have a common ancestor in the previous generation can be computed in two steps. The first stem cell picked must be derived from the type I stem cell division in the previous generation and the probability of picking it is 2α/(2α+β). Secondly, the other sampled stem cell must be the pair of the first picked stem cell in the type I division and the probability of picking it is 1/(N_t_−1). Thus the probability of finding a common ancestor (i.e. a coalescence) in a single generation backwards in time for two stem cells is:
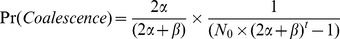
(1)The number of stem cells in intestinal crypts is approximately constant. We thus assume that 2α+β = 1 and N_t_ = N_0_ = N. Then, the above probability can be rewritten as 2α/(N−1). Once we have this single-step probability, other quantities such as the time to the most recent common ancestor for two cells and the coalescent relationship in a sample can be derived following the n-coalescent approach (Materials and Methods) [31], [32]. In this parameterization, both cell asymmetry and population asymmetry are special cases of a general model. For example, strict cell asymmetry will correspond to cases where β = 1. This general framework will allow us to test and pick the best models based on empirical observations.

### A two-deme population-genetic model for intestinal crypts

Each intestinal subunit is composed of two parts: a protrusion compartment called villus, which contains terminally differentiated cells, and an invagination compartment named crypt, which hosts stem cells and highly proliferative transit-amplifying cells. There is a continuous process that replaces functional cells in the villi with cells grown out of the crypts. We used a two-deme population-genetic model to capture continuous renewal of stem cells and transit-amplifying cells (Figure 1A). In each generation, the stem and non-stem cell population follow a dynamic process as described in the previous section. The only exception is that differentiated descendants from the stem cell deme (population 1) are constantly migrating into the other population (transit amplifying cell deme) (Figure 1B). One-way migration in the two-deme population model reflects the coupling of the stem and non-stem populations in the intestine.

In practice, when we take a random sample of cells from a crypt, we do not know whether they are stem or differentiated cells. Thus, the number of sampled cells from two demes (denoted as (m, n)) will follow a hypergeometric distribution (N_1_, N_2_, m+n), where N_1_ and N_2_ are population sizes for the two demes. Given (m,n) cells from deme 1 and deme 2, the coalescent process of going backwards in time and finding common ancestors for these lineages can be modeled using a Markov Chain (Figure 1B and 1C). The transition probability between neighboring states can be calculated as a combination of individual coalescence or migration events. For example, a single coalescence and one migration event in deme 2 will change the state from (m,n) to (m+1,n−3) (Figure 1C). For the sake of computational efficiency, we only consider transitions involving a maximum of two events (either coalescence or migration). Probability of three events occurring in one transition is low and is neglected in our calculations (Text S1). Similar to previous studies [34], when we compare the Markov Chain results with exact calculations performed through forward simulations, we find that approximate results provide an accurate characterization of the underlying dynamics (Figure 1D, Materials and Methods). Thus, a two-deme population-genetic model and the associated coalescent process can be used to model dynamics underlying cellular homeostasis of the intestinal crypt.

### Likelihood calculation and Monte Carlo Integration

Given observed genotype information (e.g. microsatellite markers, denoted as D), obtained by assaying single cells, the likelihood of the data can be calculated as
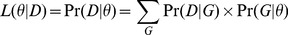
(2)where θ is the set of model parameters, including symmetric/asymmetric division parameters (α, β) and the population size parameters (N1,N2). The G (i.e. gene genealogy) represents coalescent relationships in a sample of cells. In Eq.2, the Pr(D|G) can be computed using standard phylogenetic methods and the pruning algorithm can be employed to evaluate this term [35]. The Pr(G|θ) can be calculated from the coalescent process using a Markov Chain or forward simulation (see the following sections). Since we do not directly observe the underlying gene genealogy, we need to take into account all possible ancestral relationships that are compatible with the data in order to evaluate the likelihood. By integrating over all these genealogies, the likelihood of observed data can be calculated as a function of the underlying parameters (Eq.2).

In practice, given the large dimensionality of genealogical spaces, it is not feasible to exhaustively explore all possible ancestry relationships in a sample. Instead, we use a Monte-Carlo approach to compute the likelihood in Eq.2:
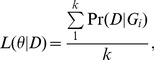
(3)where G_i_ is sampled from Pr(G |θ). It can be shown that, as k increases, likelihoods from Eq.2 and Eq.3 will converge to the same value in the limit [36].

Sampled gene genealogies can be drawn either from the Markov Chain or forward simulations depending on whether a cell population has reached equilibrium (i.e. stationary distribution) at the time of data acquisition. Markov Chain calculations assume that a given population has reached equilibrium under a given configuration of symmetric/asymmetric division rates, which may not always be true. On the other hand, forward simulation can be applied to either non-stationary or stationary scenarios, but is computationally much more expensive.

Through computational simulations, we found that stationarity has been reached for samples taken on day 340 across most of the parameter space, but not on day 52 (Text S1). Therefore, we generated genealogies for those non-stationary scenarios by simulating a crypt population history with a phase of crypt morphogenesis, followed by a period of homeostatic renewal (Materials and Methods) [25]. The generation number for the associated time point is calculated as the number of cell divisions within a given amount of time. For example, the stem cells are dividing at a rate of about once every 22 hours [25] (Materials and Methods). Since genealogical relationships are directly recorded during the course of the computer simulation, simply picking a sample of cells at the end of a simulation run yields a sample from the distribution of gene genealogies.

After averaging over many possible genealogical histories, we can calculate the likelihood of the observed data (i.e. microsatellite markers). Given the likelihood function, maximum likelihood approaches can be employed to infer the most likely parameter values. In particular, we are interested in estimating the proportion of symmetric/asymmetric divisions (α, β) in the life history of mice.

### Single-cell data and the statistical inference

Single-cell genotype data were taken from a previous study [28]. Two mice from a DNA repair deficiency strain (Mlh1−/−) with much elevated mutation rates [28], [29] were sacrificed at two different ages (day 52 and 340). From each mouse, two crypts were harvested from the mouse colon. Multiple cells (4–6, Table 1) were subsequently isolated from each sample and sequenced at a set of micro-satellite markers.

**Table 1:** Maximum-likelihood estimates asymmetric division rate.

Time	ID	n	β^a^	lnL	β^b^	lnL
Day 52	Crypt1	6	0.76	−305.12	0.76 (0.237, 1.0)^c^	−685.41
	Crypt2	5	0.60	−380.21		
Day 340	Crypt1	5	0.00	−327.46	0.00 (0, 0.441)^c^	−734.29
	Crypt2	4	0.00	−406.82		

a : estimated for each crypt.

b : estimated for each mice.

c : confidence interval calculated from the non-parametric bootstrap samples.

**Figure 2:**
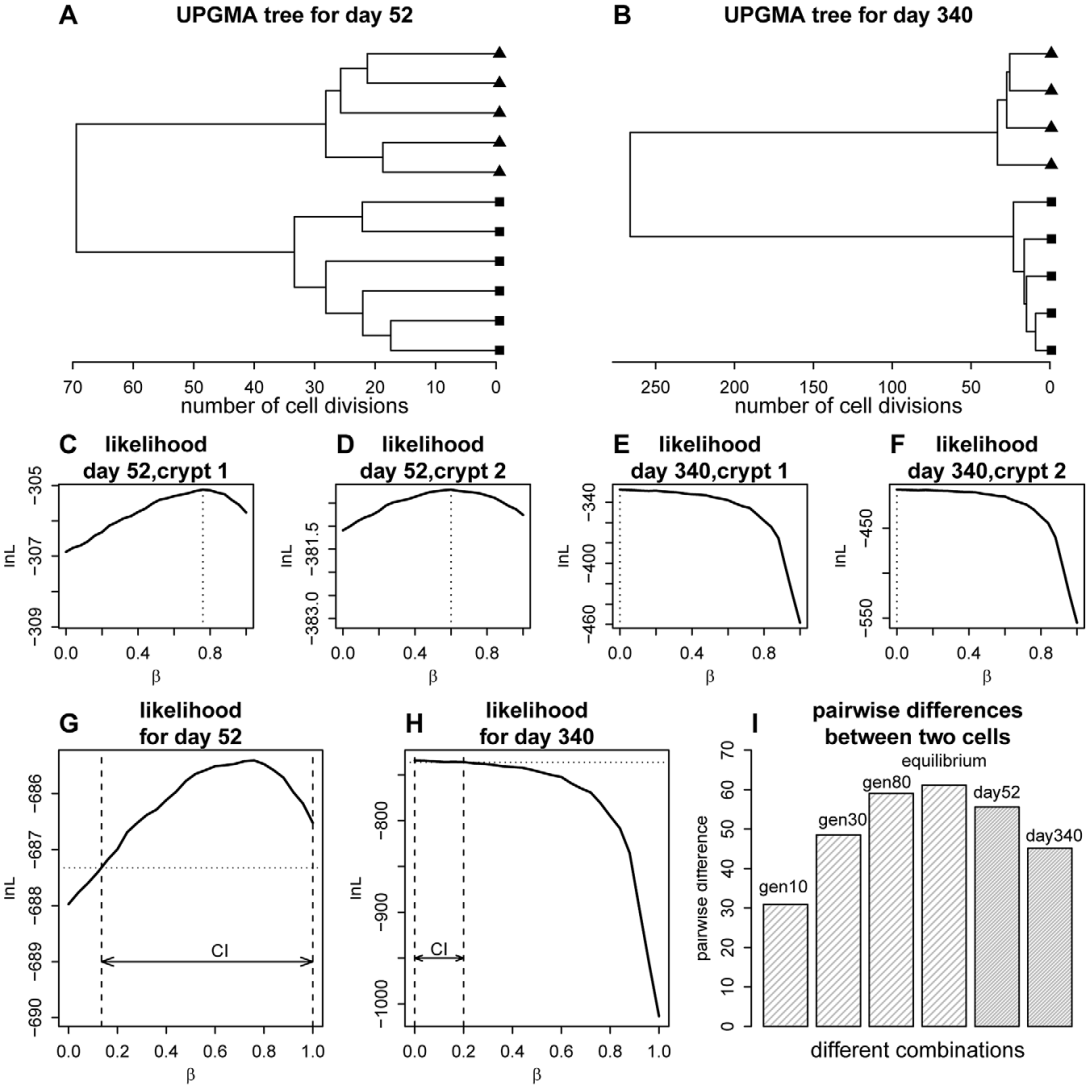
Phylogenetic relationships for the crypt single cells and likelihood curve for the proportion of asymmetric divisions. (A) Unweighted Pair Group Method with Arithmetic mean(UPGMA) tree [66] for the cells sampled from the two crypts at day 52. Distances were calculated using the two-step mutation model. (B) UPGMA tree for day 340. (C) Likelihood curve as a function of the proportion of asymmetric divisions for one of the crypts at day 52. (D) The same plot as panel C, but for the other crypt at day 52. (E) Likelihood curve as a function of the proportion of asymmetric divisions for one of the crypt at day 340. (F) The same plot as panel E, but for the other crypt at day 340. (G) Likelihood curve as a function of the proportion of asymmetric divisions for day 52 (combining two crypts). The horizontal dashed line marks the level of likelihood that is 1.92 units (0.5×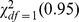) below the maximal value. The confidence interval (CI) for the proportion of asymmetric division rate is shown as the arrow between the two vertical dashed lines. (H). Likelihood curve as a function of the proportion of asymmetric divisions for day 340 (combining two crypts). The horizontal dashed line marks the level of likelihood that is 1.92 units below the maximal value. The confidence interval (CI) for the proportion of asymmetric division rate is shown as the arrow between the two vertical dashed lines. (I) A demonstration of the phenomena that the gene tree will increase in size as the crypt population reaches equilibrium (stationarity). The genealogical tree size (indicated as the mean pairwise divergence for two randomly picked cells) at different cell generation (generation 10, 30, 80 as well as equilibrium point) for the crypt population are shown (β = 0.4). The observed pairwise divergence for day 52 and day 340 are also plotted.

Using a two-step mutation model for micro-satellite markers, we first calculated the genetic distances between all sampled cells (Materials and Methods). As shown in Figure 2, individual cells within an intestinal crypt are monophyletic and are clonally related. Between-crypt divergence rapidly increases with age (Figure 2B), in agreement with previous observations of fast clonal turnover in intestinal crypts [23].

Using the likelihood approach we outlined above, we calculated the likelihood of the data as a function of asymmetric division rate for each crypt (Figure 2C–2F). For example, using the two crypts sampled from day 52 mice, maximum-likelihood estimates for the proportion of asymmetric divisions are 0.76 and 0.60 respectively (Figure 2C–2D). Interestingly, when we look at stem cells from the older mice (day 340), maximum-likelihood estimates are 0, which means that stem cells are all dividing symmetrically (Figure 2E–F). When we combined the data from both crypts at each life stage, maximum-likelihood estimates for proportions of cells dividing asymmetrically at day 52 and 340 are 0.76 and 0.0 respectively (Figure 2G–H). Our analyses thus suggest that the stem cell populations have changed from largely asymmetric divisions to solely symmetric ones.

Even though the point estimates of the asymmetric division rate is rather different, the confidence in the point estimates is not very strong. This is especially true for the day 52 (Figure 2G). In order to assess the uncertainties in the point estimates, a resampling-based nonparametric bootstrap analysis was conducted [37] (Materials and Methods). As shown in Table 1, the estimates for the asymmetric division rates at these two time points stay quite disparate, even though the confidence intervals overlap with each other. In order to compare these two point estimates rigorously, we explicitly tested the null hypothesis of H_0_: β_52_ =  = β_340_ against the alternative hypothesis H_a_: β_52_≠β_340_. Since the null hypothesis is a special case of the alternative hypothesis (i.e. the models are nested), a Likelihood Ratio Test (LRT) can be employed to ask whether the null hypothesis can be rejected with confidence. After we calculated the associated test statistic, the LRT gives the p-value of 0.024, which is significant at the nominal cut-off of 5% (Table 2). Statistical significance is also observed when we compare β_52_–β_340_ with zero over the bootstrap samples (Figure S1, P = 0.04). In other words, there is strong statistical evidence that the proportion of cells undergoing asymmetric divisions is very different between day 52 and 340.

**Table 2:** Likelihood ratio tests under different models.

Model	lnL under H_0_	lnL under H_a_	−2ΔlnL	pvalue
Two deme model (Discrete)	−1422.26	−1419.69	5.13	0.024*
Age structure model (Discrete)	−1442.97	−1430.57	24.81	6.3×10^−7^ **
Spatial model (Discrete)	−1434.66	−1425.16	19.02	1.3×10^−5^ **
Continuous time models	−1417.76	−1415.39	4.75	0.029*

* : significant at 5% level,

** : significant at 1% level.

In addition to the two-deme model outlined above, we explored a series of more complicated models that reflect various additional aspects of crypt biology. In general, due to complexity of these models, analytical results are much harder to derive, but can be supplemented with computer simulations (Materials and Methods). For example, when we compute the log-likelihood under a model where the population of the transit-amplifying cell pool is age-structured (Table 2, Figure S2), the likelihood ratio test shows even stronger evidence of differences in asymmetric division rates between day 52 and 340 (P = 6.3×10^−7^). This is also true when we explore spatial structures of the intestinal crypt (P = 1.3×10^−5^, Table 2, Figure S2), as well as continuous-time models where waiting times between events are exponentially distributed and population sizes are allowed to fluctuate within a certain range (P = 0.029, Table 2, Figure S3). In addition, we also examined possible variations in mutation rates and found that the results stay qualitatively similar (Materials and Methods, Text S1). In summary, the conclusion that stem cells transition from asymmetric to symmetric division is insensitive to model details.

## Discussion

Using population genetic theory, in particular the coalescent theory, we have drawn an extraordinary dynamic picture of intestinal crypt homeostasis. Compared to earlier lineage-tracing methods which typically do not allow for individual lineage relationships, this branch of theory provides a more detailed picture of stem-cell crypt dynamics. With single-cell data collected from different life stages, we found strong statistical support for a transition from cell asymmetry to population asymmetry during mouse life history.

Intuitively, the reason we can observe this discrepancy is that genealogical trees of crypt cell populations will steadily increase in size as the population evolves to establish equilibrium. This is analogous to the founder population effect in population genetics (Figure 2I). The genealogical trees for day 52 (before equilibrium) are expected be much shorter than those from later times if asymmetric division parameters are constant. However, the gene trees at day 52 are observed to be larger than those at day 340 (Figure 2I). The discrepancy between expectation and real observation leads to the inference of higher asymmetric division rate at the early life stage, because asymmetric division will slow down stem cell lineage turnovers and increase genealogical tree size.

**Figure 3:**
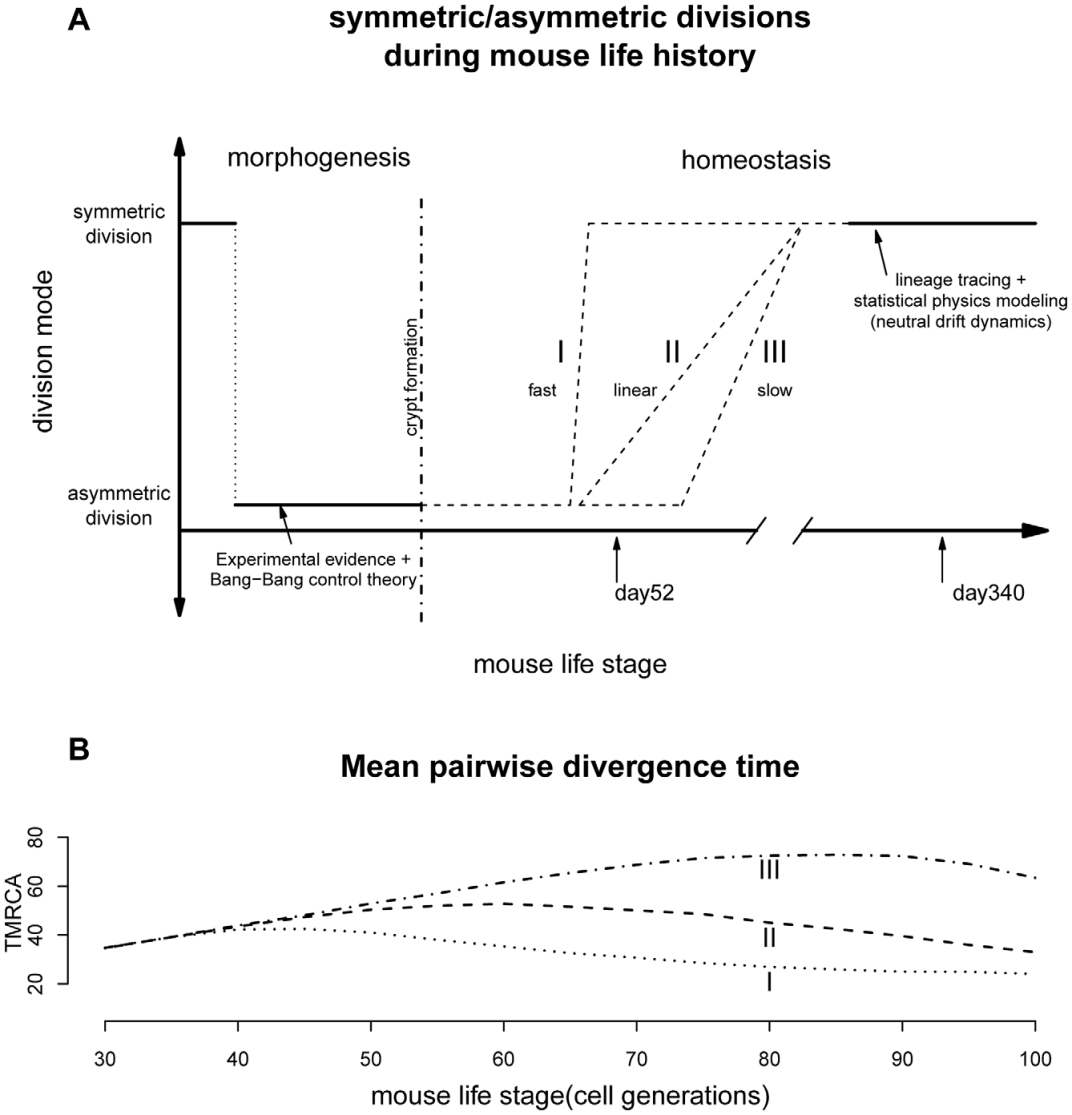
Lifelong equilibrium for stem cell symmetric/asymmetric divisions. (A) The proportion of asymmetric and symmetric division as a function of mouse life stage. The pattern from morphogenesis are from experimental data together with the optimal control theory prediction [25]. The lineage tracing evidences are from two earlier studies [23], [24]. The transition from cell asymmetry to population asymmetry can take a variety of forms, including I) a fast switch similar to the Bang-Bang control, II) a linear accumulation, III) slow progression depending on the interplay between intrinsic and extrinsic signals. (B) When simulating crypt histories using curve I, II and III in Figure 3A and plotting mean pairwise divergence time between two random cells (a measurement of genealogical length), the average pairwise divergence shows very different trajectories under different transitional history.

Previous observations revealed that mouse crypt morphogenesis started with a surge of symmetric divisions establishing the pool of stem cells, followed by a transition to predominantly asymmetric divisions that maintain an equilibrium between stem cell self-renewal and differentiation [25]. Our results support the existence of a second transition from mostly asymmetric stem cell divisions to symmetric divisions during intestinal homeostasis (Figure 3A). It remains to be seen whether this phenomenon also occurs in other systems.

The population asymmetry found here for day 340 matches previous observations that adult intestinal stem cells are maintained by replacing randomly-lost cells through predominately symmetric divisions of their neighbors (Figure 3A) [23], [24]. This random neutral drift also allows monoclonal turnover observed previously [28]. The stochastic fate determination of stem cells seems to be quite general across many tissue types and species [13], [14], [38].

Our observations raise a number of questions about the dynamics of the transition between cell division modes. Stem cell behaviors are often controlled by both internal signals (e.g. cellular polarity [39] or telomerase activity [40]) and external factors (e.g. BMP pathway in the mesenchyme) [16], [18], [41]. What are the relative roles of these intrinsic and extrinsic factors are still not fully understood and therefore the exact molecular mechanisms behind the transition between cell division modes are still unknown.

Furthermore, transition timing is also unclear. If paneth cells are responsible for maintaining much of the intestinal niche [42], the transition might be quite fast since stem niche and stem cells are derived from the same cell cascade where an upstream triggering signal can easily be propagated downstream and the transition can be hastened through a snow-ball-like effect (Figure 3A). On the other hand, if signals from other mesodermal components (e.g. mesenchyme) are also contributing to this transition, we might imagine the stem cell and their niche could be changing asynchronously leading to a variety of histories with drastically different paces [43], [44].

Interestingly, current biological evidences seem to have a tilt towards a fast transition. For example, lineage tracing study looking at the speed of drift towards monoclonality, has found similar rates for mice of age 1.5, 6.5 as well as 8 months [23]. In other words, starting from about 45 days, the crypt dynamics could potentially have shifted to largely symmetric divisions. In our model we were treating asymmetric/symmetric parameter as a fixed unknown constant and the coalescent analysis is a retrospective approach looking at the profile of a recent history before the time of observation (typically within 4N generations, where N is the population size [30]). Our results thus reflect a time-average measurement. The fast transition might have lead to the statistical uncertainties for the asymmetric division rate observed for day 52. Nevertheless, based on our limited simulations with time-varying asymmetric division rates, changes in transition dynamics should leave very different genealogical signatures, where future studies with dense sampling across time will be able to resolve this landscape more precisely (Figure 3B). After all, the pattern observed here seems to suggest that the switch has started not long before day 52, which is broadly around mouse maturation (Figure 3 and following sections).

Why do the crypt cells need to switch to population level asymmetry, which is an apparently more fragile scheme for long-term tissue maintenance [10]? There are two possible explanations for this transition. One explanation involves acquisition of the population asymmetry driven by adaptive mechanisms. Asymmetric divisions may be a harder task for cells to perform as cell fate determinants all need to be delivered to the two daughter cells according to the two distinct states [5]. In contrast, the easier mode may be symmetric divisions in which the two daughter cells need not be distinguished. The implication for the transition is that as mice grow old, their cells gradually take the easier mode. In addition, since stem cell function often declines with age, population asymmetry might allow stem cells to effectively repair and restore homeostasis – a key adaptation that can increase capacity for repair and increase life-span [45]. Furthermore, adaptive immune response to environmental insults including gut microbiota infection can also possibly contribute to the homeostatic transition [44], [46], [47]. In light of this view, cancer and tissue loss, two flip sides of normal cellular dynamics, might result from disruptions of this cell division equilibrium [5], [48].

On the other hand, there might also exist a “passive” explanation for this transition. The observed progression can simply be a by-product of natural selection. When a single gene has multiple functions, some of the functions will be beneficial to the organism, while others might be detrimental. Most importantly, when the advantageous gain outweights the deleterious costs, the target gene can still be selected (i.e. antagonistic pleiotropy) [49]. During the course of evolution, a gene with multiple effects will be strongly optimized for its function before reproduction, and as a consequence also produce deleterious effects in later life [49]. In this light it is notable that the transition in cell division mode occurs roughly at the time of sexual reproduction (Figure 3). Many proteins involved in asymmetric cell divisions also function as tumor suppressor genes [5], [50], [51]. The loss of asymmetric division might thus simply be driven by the increasing need for active tumor suppressors [52].

The life-history of stem cell division is not yet fully discernable from our results (e.g. Figure 3). For example, our estimate of the proportion of asymmetric divisions for day 52 is still quite high, even though we have evidence that the proportions at days 52 and 340 are significantly different. Information collected from only two time points also prevents sophisticated models where many of the parameters can be treated as time-varying variables rather than fixed constants. Future studies with denser serial sampling together with larger number of crypts might be able to draw a more concrete picture of the crypt homeostasis.

Until now, asymmetric divisions were thought to be rarer in vertebrates than invertebrates [5]. However, new evidence for the existence of this mode is starting to accumulate for a few tissue types such as the central nervous system [53], skin [54], [55], the hematopoietic system [51], [56], [57] as well as the intestinal crypts [26]. Since the mechanisms controlling stem cell symmetric and asymmetric divisions are often conserved across the tree of life [16], the pattern observed here for the mouse intestine is very likely to be quite general. With the advances in genomic technology, in particular whole-genome single-cell sequencing [58], [59], we should be able to reveal a more lively cellular orchestra across a wide range of organ types and species, each with its own mechanism and equilibria.

## Materials and Methods

### Population dynamics for cells within intestinal crypts

Since population sizes are relatively constant in the intestinal crypt and because type III divisions do not change the number of stem cell descendants, the proportions of two types of symmetric divisions (type I and II) have to be balanced and are set to be equal so that the total number of cells is maintained (Figure 1A). In other words, 1-α-β (proportion of type II divisions) is be equal to α (proportion of type I divisions). In each generation, fraction α of the stem cells divides symmetrically, each cell giving rise to two stem cells (type I); fraction β divides asymmetrically (type III) and fraction α divides symmetrically with each cell producing two differentiated cells (type II). Differentiated cells from both asymmetric and symmetric divisions migrate to the transit-amplifying cell pool.

Since there is a constant extrusion of cells from the crypt into the villi, we capture the dynamics of the transit-amplifying cell pool by allowing only a certain proportion of the cells to participate in reproduction for the next generation. The remaining cells are extruded out of the deme 2. We set the population size of stem cells (population 1) to N1 and that of transit-amplifying cells (population 2) to N2. In each generation there are N1 cells migrating to the transit-amplifying cell pool. In the transit-amplifying cell population, fraction γ of N2 cells divide once and give rise to two descendant cells. The remaining (1−γ)×N2 cells are extruded outside of the population 2. The value of γ is set to (N_2_-N_1_)/2N_2_ such that the population size in population 2 is constant. Based on previous observations in mouse colon crypts [60], [61], we set the population size N1 and N2 to be 15 and 185. Population sizes such as 250 (15 stem and 235 non-stem cells) are also tried and results stay similar.

### Genealogical histories, Markov Chain, and the forward simulation

Given the number of cells we sampled in two demes, the process of going backwards in time and finding common ancestors can be modeled as a Markov Chain. The state transitions are given by combinations of individual coalescence or migration events. For two randomly sampled lineages, the expected time to their most recent common ancestor (MRCA) can be computed by directly simulating from a Markov Chain following the appropriate state transitions. The expected value for the time to MRCA for two lineages can also be derived analytically using a first-step analysis of a given Markov Chain (Figure 1D). In Text S1, we presented the details of the transition probabilities between state spaces for the Markov Chain.

The Markov Chain calculation assumes that data are collected from a stationary process. Based on our simulations, we find that, for most of the parameter values, stationary distributions have been reached by day 340. However, this does not appear to hold for day 52 (Figure S4 and Text S1). In these cases, forward simulations are used to generate genealogical histories from Pr(G|θ). To achieve this, we simulated population histories with two phases: morphogenesis and homeostasis (Figure 3). This scenario was chosen to reflect experimental evidence as well as predictions from the Bang-Bang control theory [25]. In this process, the intestinal crypt is founded by first creating N1 stem cells, followed by a series of asymmetric divisions to generate the transit-amplifying cell population. After crypt morphogenesis, populations follow the dynamic process described in the previous section. At the end of our simulations, a random subset of cells is sampled and their genealogical history is recorded (see Text S1 for details).

To estimate the number of generations leading to day 52, we tried a series of approaches. Since we know that crypt morphogenesis starts around post-natal day 7 for mice [62], [63] and stem cells divide every 22 hours [24], [25], postnatal day 52 corresponds to about generation 50 starting from crypt morphogenesis (roughly 42 generations after crypt formation because crypt morphogenesis takes around 8 generations). Because the exact cell generation number will be a random variable around this mean value, we used various forms of random distributions (e.g. beta distribution or truncated normal distribution) to model the generation number. Conditioning on the generation number, gene genealogies in the forward simulation at the corresponding generation number is sampled for the Pr(G|θ) at day 52. (see Text S1 for details).

### Single-cell data acquisition

Data were taken from a previously-published study [28] where multiple single-cell genotypes were sequenced from a DNA-repair deficiency mouse strain (Mlh1−/−). This strain has elevated mutation rates, allowing for enough informative mutations to enable our analyses [28], [29]. Two mice at day 52 and 340 were sacrificed. From each mouse, two crypts were harvested from the mouse colon. Multiple cells (4–6, Table 1) were then isolated and sequenced at a set of micro-satellite markers. In total, 150 microsatellite markers were genotyped, from which only markers with successful genotyping information from at least two cells were extracted for this work (70–90 markers).

### Alternative models and genealogical histories

We also tried a series of alternative models to explore other aspects of stem cell dynamics.

In the age structure model (Figure S2A), multiple demes exist in the transit amplifying cell population. Each of these demes corresponds to cells with different ages (number of divisions since leaving the stem cell deme). Cells hitting an age limit will be extruded out of the crypt. In the spatial model (Figure S2B), multiple spatial demes corresponding to cells at different localities are constructed. Demes in the transit amplifying cell population have different probability of being extruded from the crypt depending on their physical locations.

In the continuous-time models, the time to the next event (waiting time) is exponentially distributed with the intensity parameter specified by the cell division rate (λ). The time to the next event for n cells is exponentially distributed with rate nλ. Given the time to the next event, the exact cell that experiences this event is randomly picked among the n cells following statistical properties of the Poisson Process [64]. When a stem cell is picked, the possible events are type I/II/III stem cell divisions, depending on the values of α and β. If a transit-amplifying cell is picked, it either divides or is extruded out of the crypt with the associated probability. In this simulation, we allowed the stem cell population to fluctuate within a size range (size from 10 to 20 with a mean of 15), a feature we implemented using rejection sampling [64]. The details of these models are presented in Text S1.

In general, analytical results from these models are much harder to derive, but computational simulations can be conducted to sample gene genealogies from the random process. Likelihood calculations follow the same procedures as previous models after sampling gene genealogies from Pr(G| θ).

### Pruning algorithm, the mutational model, and the UPGMA tree

Given a gene tree, the likelihood of the observed data can be computed from the tip of the tree towards the root using the pruning algorithm [35]. For the microsatellite loci, we used a two-step mutational model where repeat number j can mutate to j±1 and j±2. Previous empirical measurements have found that the average mutation rate is 0.01 per site per generation and size-two transitions (j±2) are happening at 1/7 the frequency of size-one mutations (j±1) [28]. We also explored other mutational models and they did not affect our conclusions (data not shown).

In order to further explore the possibility of mutation rate variation, we used various forms of the beta distribution to capture uncertainty in the mutation rate. Since the mutation rate measured from previous studies is 0.01 per site per generation, we adopted beta distributions with different shape parameters, but transformed to take values between 0.0075 and 0.0125. The likelihood of the data can be calculated by partitioning the mutation distribution into discrete bins and taking the weighted sum of individual likelihoods calculated at discrete values of mutation rates [65] (see Text S1).

Unweighted Pair Group Method with Arithmetic mean (UPGMA) tree [66] was built using functions implemented in the APE (Analyses of Phylogenetics and Evolution) library [67] within the R package (http://www.R-project.org). The pairwise distances between the cells were calculated using the mutation matrix specified in the previous section.

### Statistical inference

The likelihood of the data as a function of the underlying parameters can be computed in a Monte Carlo fashion as in Equation 3. We sampled 20,000 gene genealogies to compute the log-likelihood for each combination of parameter values. A non-parametric bootstrap test was conducted by resampling microsatellite markers from the original dataset with replacement (100 replicates) and re-running the analyses. In the likelihood ratio test, the maximum-likelihood values under the null and alternative model respectively are extracted. Likelihood Ratio Test (LRT) is conducted by comparing twice the log-likelihood ratio to the chi-square distribution with one degree of freedom, since the two models we compared were nested with the alternative having one extra parameter.

## Supporting Information

Figure S1 The distribution of estimated β_52_–β_340_ over the bootstrap samples. For each re-sampled bootstrap datasets, we can get an estimate of β_52_ and β_340_ respectively. When we take a difference between the two point estimates and plot its distribution, we get the histogram shown in this figure. The number of replicates with β_52_–β_340_ less or equal to zero is 4 (out of 100).
(PDF)

Figure S2 Alternative models exploring additional aspects of the cellular dynamics within intestinal crypts. (A). In the age-structure model, there are multiple demes with different ages in the non-stem cells. In each generation, non-stem cells in deme i (age i) migrate into deme i+1. Non-stem cells reaching a maximum age (denoted as K) will be extruded out of the crypt in the next generation. (B). In the spatial model, multiple spatial demes exist in the non-stem cell pool. In each spatial deme, non-stem cells have a certain probability of staying in the original deme and with remaining probability of moving to the next deme or being extruded out of the crypt. The exact setup of these two models is presented in great detail in the Text S1.
(PDF)

Figure S3 The likelihood of the data under different distributions for mutation rates. (A) Log likelihood profile for the first crypt at day 52 under different beta distributions. (B) The same plot, but for the second crypt at day 52. (C) Log likelihood profile for the first crypt at day 340. (D) The same plot, but for the second crypt at day 340.
(PDF)

Figure S4 Mean pairwise divergence time between two cells at different cell generations for different asymmetric/symmetric division rates. The X axis is the generation time and the y axis is the mean pairwise difference. (A) beta = 0, (B) beta = 0.2, (C) beta = 0.4, (D)beta = 0.6, (E) beta = 0.8.
(PDF)

Text S1 1) The derivation for state transitions in the Markov chain. 2) Alternative models and their setup. 3) Crypt history and genealogical sampling at day 52. 4) Mutation rates and the likelihood calculation.
(DOC)
