# Supplementary figures and images for: Age-Dependent Transition from Cell-Level to Population-Level Control in Murine Intestinal Homeostasis Revealed by Coalescence Analysis
Source: PLoS Genet. 2013 Feb 28;9(2):e1003326. doi: 10.1371/journal.pgen.1003326 (PMC3585040; doi:10.1371/journal.pgen.1003326)

**difference in asymmetric division rate  
over 100 bootstrap samples**

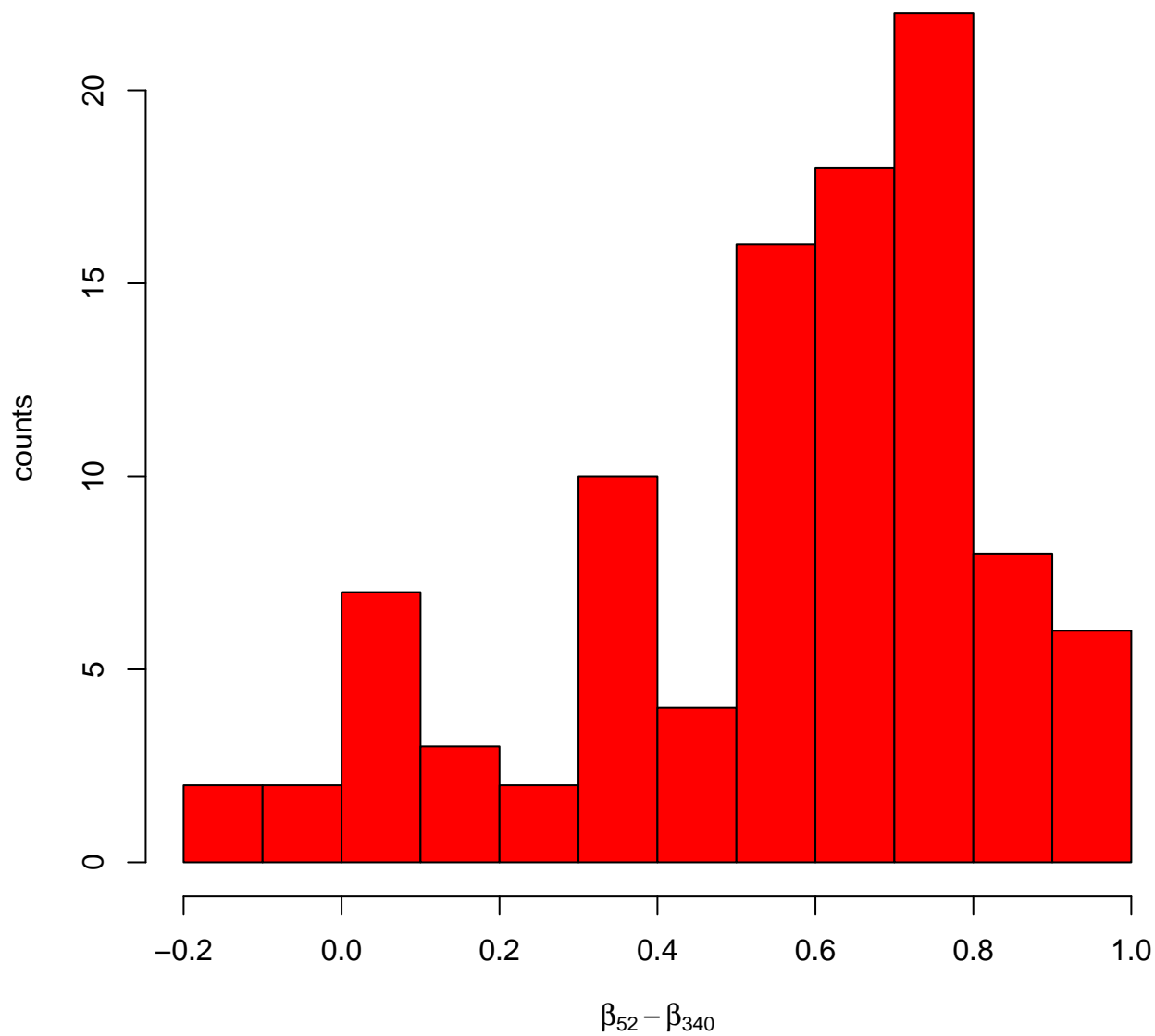

Supplement: Figure S1 — The distribution of estimated β52–β340 over the bootstrap samples. For each re-sampled bootstrap datasets, we can get an estimate of β52 and β340 respectively. When we take a difference between the two point estimates and plot its distribution, we get the histogram shown in this figure. The number of replicates with β52–β340 less or equal to zero is 4 (out of 100). (PDF) [file pgen.1003326.s001.pdf]

**A. Age-structured model**

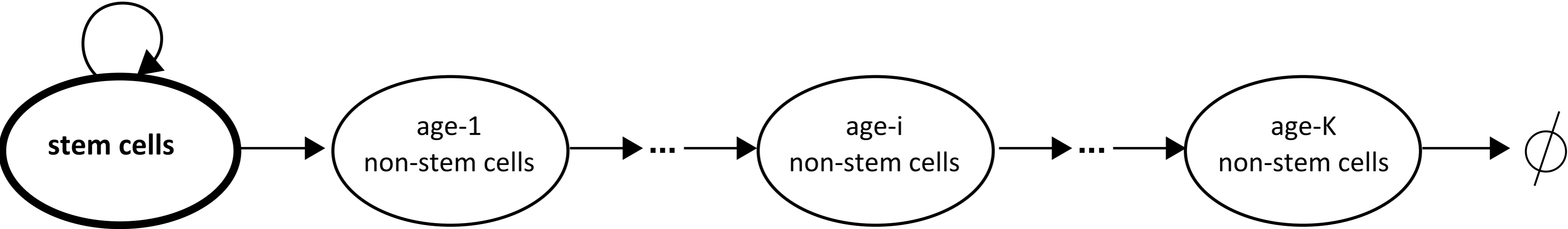

**B. Spatial model**

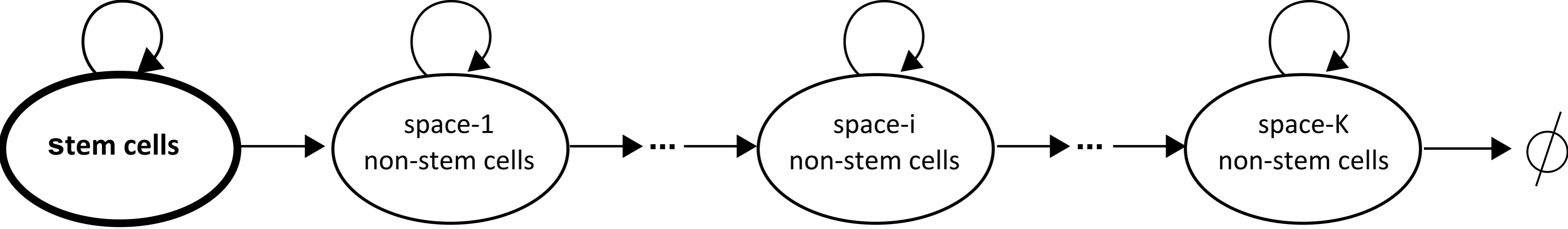

Supplement: Figure S2 — Alternative models exploring additional aspects of the cellular dynamics within intestinal crypts. (A). In the age-structure model, there are multiple demes with different ages in the non-stem cells. In each generation, non-stem cells in deme i (age i) migrate into deme i+1. Non-stem cells reaching a maximum age (denoted as K) will be extruded out of the crypt in the next generation. (B). In the spatial model, multiple spatial demes exist in the non-stem cell pool. In each spatial deme, non-stem cells have a certain probability of staying in the original deme and with remaining probability of moving to the next deme or being extruded out of the crypt. The exact setup of these two models is presented in great detail in the Text S1. (PDF) [file pgen.1003326.s002.pdf]
