# Supplementary material for: Age-Dependent Transition from Cell-Level to Population-Level Control in Murine Intestinal Homeostasis Revealed by Coalescence Analysis
Source: PLoS Genet. 2013 Feb 28;9(2):e1003326. doi: 10.1371/journal.pgen.1003326 (PMC3585040; doi:10.1371/journal.pgen.1003326)
Supplement: Figure S3 — The likelihood of the data under different distributions for mutation rates. (A) Log likelihood profile for the first crypt at day 52 under different beta distributions. (B) The same plot, but for the second crypt at day 52. (C) Log likelihood profile for the first crypt at day 340. (D) The same plot, but for the second crypt at day 340. (PDF) [file pgen.1003326.s003.pdf]

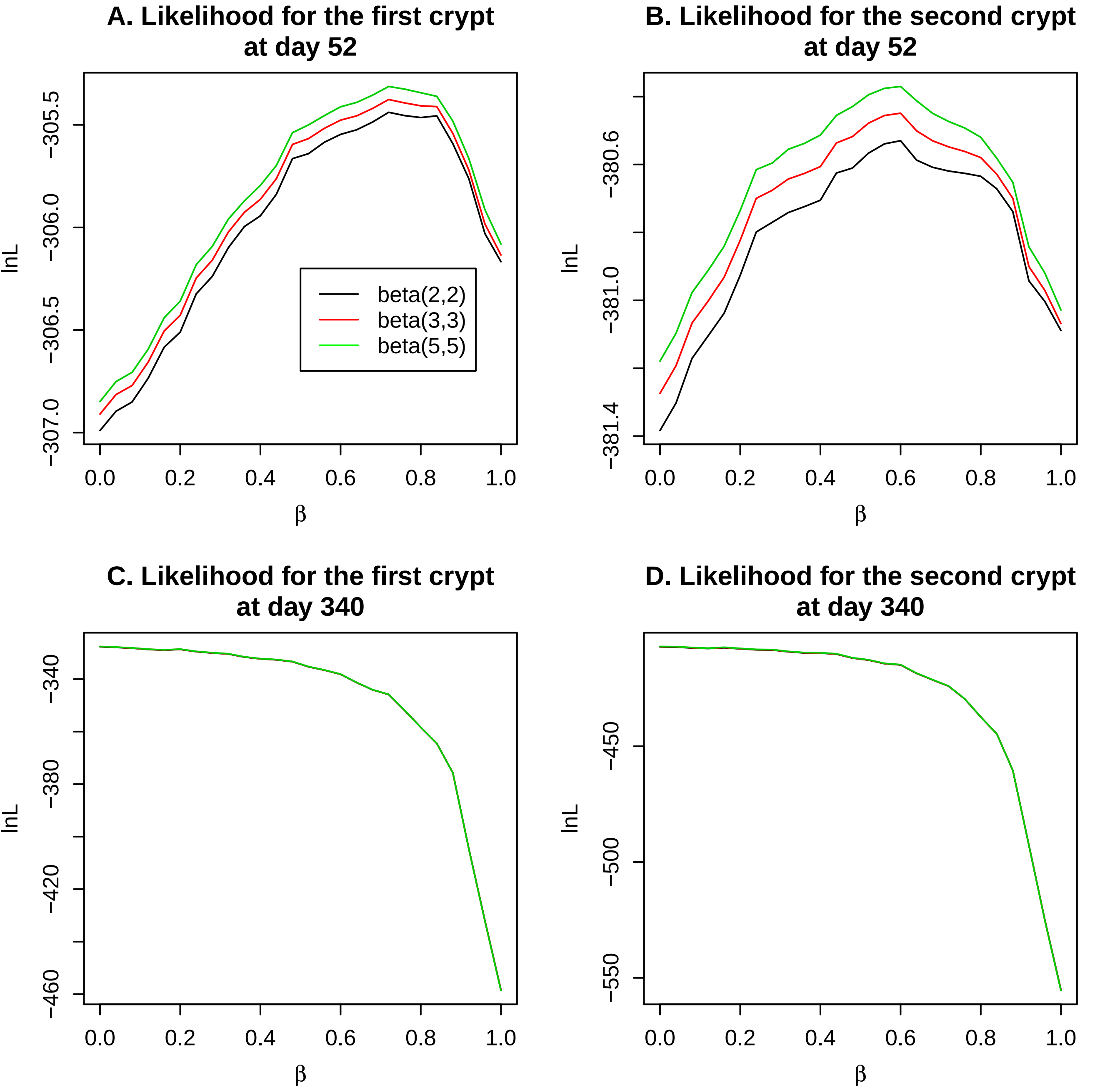

A. Likelihood for the first crypt at day 52
-305.5
-306.0
-306.5
-307.0
lnL
0.0
0.2
0.4
0.6
0.8
1.0
β
beta(2,2)
beta(3,3)
beta(5,5)
B. Likelihood for the second crypt at day 52
-380.6
-381.0
-381.4
lnL
β
C. Likelihood for the first crypt at day 340
-340
-380
-420
-460
lnL
β
D. Likelihood for the second crypt at day 340
-450
-500
-550
lnL
β
