# Supplementary material for: Age-Dependent Transition from Cell-Level to Population-Level Control in Murine Intestinal Homeostasis Revealed by Coalescence Analysis
Source: PLoS Genet. 2013 Feb 28;9(2):e1003326. doi: 10.1371/journal.pgen.1003326 (PMC3585040; doi:10.1371/journal.pgen.1003326)
Supplement: Figure S4 — Mean pairwise divergence time between two cells at different cell generations for different asymmetric/symmetric division rates. The X axis is the generation time and the y axis is the mean pairwise difference. (A) beta = 0, (B) beta = 0.2, (C) beta = 0.4, (D)beta = 0.6, (E) beta = 0.8. (PDF) [file pgen.1003326.s004.pdf]

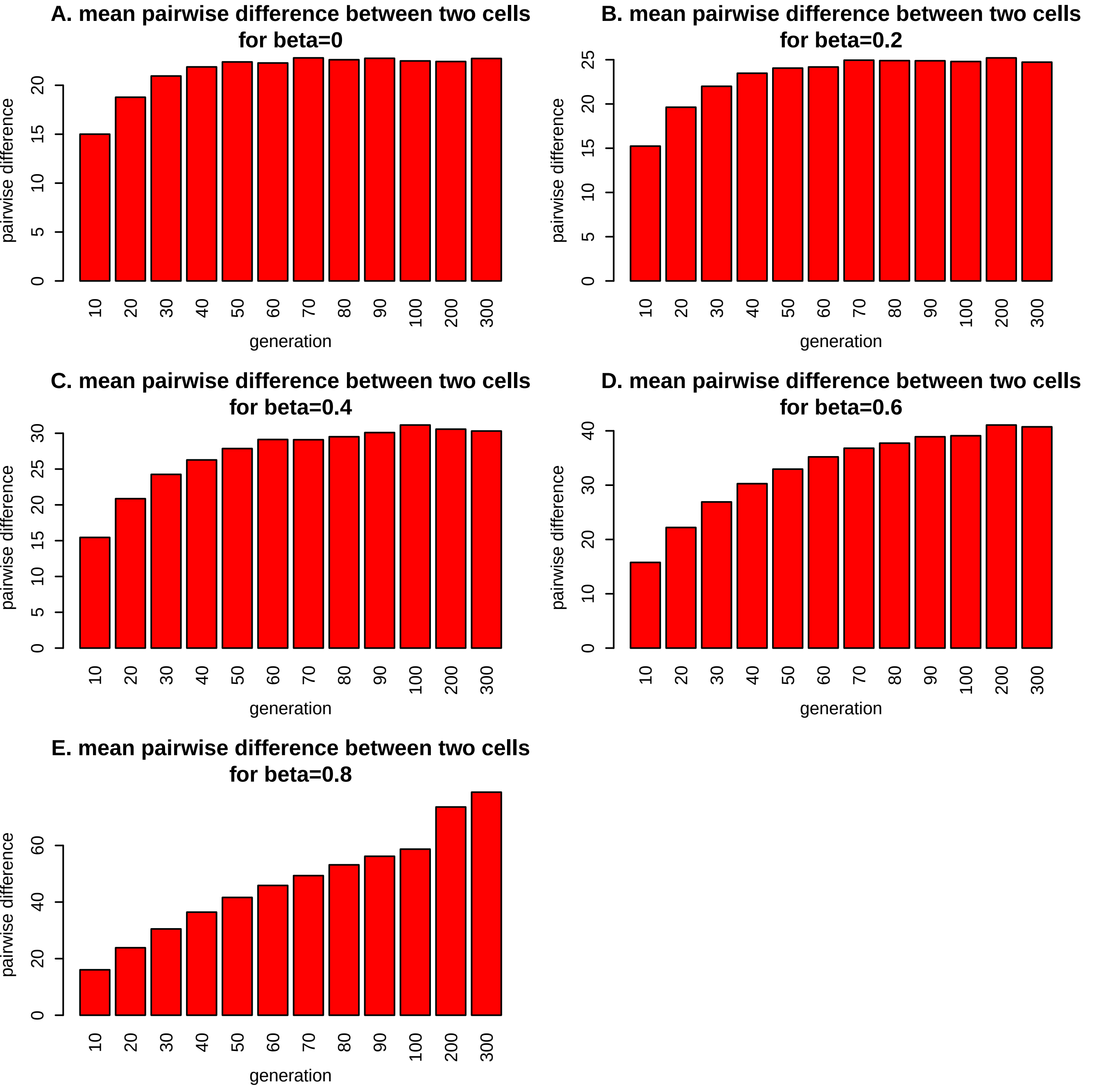
A. mean pairwise difference between two cells for beta=0
B. mean pairwise difference between two cells for beta=0.2
C. mean pairwise difference between two cells for beta=0.4
D. mean pairwise difference between two cells for beta=0.6
E. mean pairwise difference between two cells for beta=0.8
pairwise difference
generation
10
20
30
40
50
60
70
80
90
100
200
300
