# Supplementary material for: Age-Dependent Transition from Cell-Level to Population-Level Control in Murine Intestinal Homeostasis Revealed by Coalescence Analysis
Source: PLoS Genet. 2013 Feb 28;9(2):e1003326. doi: 10.1371/journal.pgen.1003326 (PMC3585040; doi:10.1371/journal.pgen.1003326)
Supplement: Text S1 — 1) The derivation for state transitions in the Markov chain. 2) Alternative models and their setup. 3) Crypt history and genealogical sampling at day 52. 4) Mutation rates and the likelihood calculation. (DOC) [file pgen.1003326.s005.doc]

**Text S1 for**

Zheng Hu^1,2^, Yun-Xin Fu^3^, Anthony J. Greenberg^4^, Chung-I Wu^1,5, ¶^ , Weiwei Zhai^1,6,¶^

^1^Center for Computational Biology and Laboratory of Disease Genomics and Individualized Medicine, Beijing Institute of Genomics, Chinese Academy of Sciences, Beijing, China,

^2^Graduate University of Chinese Academy of Sciences, Beijing, China,

^3^Human Genetics Center and Division of Biostatistics, School of Public Health, University of Texas at Houston, Houston, Texas, USA

^4^Departments of Biological Statistics and Computational Biology, Cornell University, Ithaca, NY, USA

^5^Department of Ecology and Evolution, University of Chicago, Chicago, Illinois, USA

^6^National Center for Mathematics and Interdisciplinary Sciences, Chinese Academy of Sciences, Beijing, China,

^¶^Correspondence should be addressed to: [weiweizhai@big.ac.cn](mailto:weiweizhai@big.ac.cn) and [ciwu@uchicago.edu](mailto:ciwu@uchicago.edu)

**Part one: The derivation for state transitions in the Markov chain**

In the two-deme population genetic model, we used a Markov chain to model the coalescent process when the population of interest has reached equilibrium (stationary). Here, we outlined the details of the transition probabilities.

As we discussed in the main-text, we will neglect transitions with smaller orders of probability and only consider transitions involving a maximum of two events (either coalecence or migration). For a state (m,n), there will be eight states that are communicatable with the initial state (m, n). We will outline the transition probability for each of the individual combination.


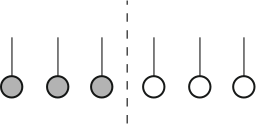
**1) (m,n) → (m,n)**

In this case, there is neither coalescence nor migration for both the stem and non-stem cell population. For the stem cell deme, there are *α×N_1_* sib-pairs of stem cells (i.e. 2×*α×N_1_* stem cells) and each pair of stem cells is derived from an ancestral stem cell in the previous generation(Type I division, Figure.1A). Likewise, *β×N_1_* stem cells are derived from an asymmetric division division (Type III division, Figure.1A).

For the m chosen stem cells to share no common ancestor in the previous generation, the m cells have to fall into distinctive stem cell division event (either type I or type III event) in producing the current generation. The number of combinations is:

,

where x is the number of stem cells that are derived from the type I symmetric divisions (Figure.1A).

For the non-stem cell deme, there are *γ×N_2_* sibling-pairs of non-stem cells (i.e. 2×*γ×N_2_* non-stem cells) and each pair is derived from a doubling event in the previous generation. Since in each generation, there will be *N_1_* non-stem cells that migrate from the stem cell deme. For the n chosen non-stem cells to have no common ancestor in the previous generation, the n cells have to fall distinctively into *γ×N_2_* pairs and the total number of combination is:

,

By summarizing the calculations in the two demes, the transition probability can be calculated as:

(1)

where the denominator is the total number of possible combinations.


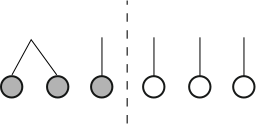
**2) (m,n) → (m-1,n):**

As depicated in the cartoon, there is only one coalescence event in the stem cell pool and there is no coalescence or migration in the non-stem cell population. The number of combinations that there is one coalescence event for the stem cells is:

where the pair of stem cells that coalecences can be any one of the *α×N_1_* pairs.

Since the number of combinations that there is no coaslescence or migration for the non-stem cells is the same as the case 1). The probability for the transition (m,n) → (m-1,n) can be calculated as:

(2)

**3) (m,n) → (m,n-1)**

**
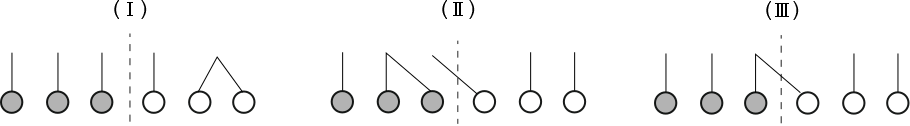
**

As shown in the above cartoon, there are three possibilities for the transition from (m,n) to (m,n-1).

(I): There is no coalescence for the stem cells and there is one coalescence, but no migration for the non stem cells. In the deme 2, the number of combinations that there is one coalescence and no migration for the non-stem cells is:

Thus the probability of case (I) for transition (m,n) to (m, n-1) is

,

(II): There is one coalecence for the stem cells and there is one migration, but no coalescence for the non-stem cells. In deme 2, the number of combinations that there is one migration and no coalescence for the non-stem cells is:

,

Thus the probability of case (II) is:

,

(III) There is one migration for the non-stem cells and the migrated non-stem cell coalesces with one of the chosen stem cell (i.e. two descendents from the type III event). In the stem cell pool, the number of combinations for the remaining m-1 stem cells to have no coalecence one generation back is:

,

In the non-stem cell pool, the number of combinations that there is one migration and no coalescence for the non-stem cells in a generation backward in time is:

,

Thus the probability of case (III) is:

In summary, the total probability of transition (m,n) → (m, n-1) is:

(3)

**4) (m,n) → (m-1,n-1)**

***
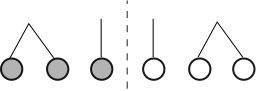
***

There is one coalescence for both stem cells and non-stem cells, and there is no migration for non-stem cells. Following derivations similar to previous cases, the probability for the transition from (m,n) to (m-1,n-1) is:

(4)

**5) (m,n) → (m+1,n-1)**

**
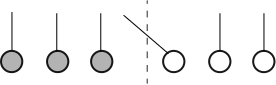
**

There is a single migration event for non-stem cells, and there is no coalescence for both stem and non-stem cells. Since the number of ancestral lineages of stem cells increases from m to m+1, the stem sibling of the migrated non-stem cell, should not be in the m stem cells chose in the stem cell deme. This is exactly the case of (III) from the transition (m,n) to (m,n-1).

Thus, the probability of transition (m,n) → (m+1,n-1) is:

(5)

**6) (m,n) → (m,n-2)**

**
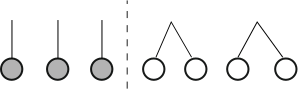
**

There are two coalescence events and no migration for the non-stem cells, and there is no coalescence event for the stem cells.

The number of combinations that there are two coalescence events and no migration for the non-stem cells is:

,

The probability for transition (m, n) → (m, n-2) is:

(6)

**7) (m,n) → (m+1,n-2)**

**
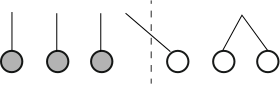
**

There is one coalescence event and one migration event for non-stem cells, and there is no coalescent event for stem cells.

The number of combinations that there is one coalescence event and one migration event for non-stem cells is:

,

Thus the probability for transition (m, n) → (m+1, n-2) is:

(7)

**8) (m,n) → (m+2,n-2)**

**
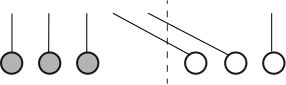
**

There are two migration events for non-stem cells, and there is no coalescence for both stem and non-stem cells.

The number of combinations that there are two migration events for non-stem cells and the two migrated cells are not siblings is:

,

Then the probability for transition (m, n) → (m+2, n-2) is: (8)

**Part two: Alternative models and their setup.**

The model construction is illustrated in Figure. S2. **A).** In the age-structure model, there is a strict age structure for the non-stem (transit-amplifying) cells. Stem cells follow the same dynamics as those in the two-deme model. Non-stem cells that just left the stem cell pool have age one. Each additional division will increase the non-stem cell from age i to i+1. When non-stem cells reach an age limit (denoted as K), the non-stem cells will be extruded out of the crypt in the next generation. We set the number of stem cells as 15 and the age limit K as 4. In order to maintain an equilibrium condition, the population sizes for the four non-stem cell demes will be 15, 30, 60 and 120 respectively. In this parameterization, the number of transit-amplifying cells is set to be 225 and total number of cells within a crypt is 240, which is consistent with an previous estimate of 200~250 cells in a crypt (See main-text).

**B).** Spatial model. The non-stem cells are partitioned into multiple spatial demes reflecting their spatial locations in the crypt rather than their ages in the age-structured model. Contrasting to the age-structure model, cells within each spatial deme have certain probabilities of staying at the original deme in each generation. In the current setting, we assumed that the 225 non-stem cells are partitioned into four spatial demes. We set the total number of cells in the first three spatial demes as 150, reflecting previous observations that around 150 cells are rapidly dividing for the non-stem cells . The remaining 75 cells in the last deme represent cells in the extrusion zone. We set the probabilities of remaining in the same deme in the next generation for the first three demes as 1/8, 1/9 and 5/170 respectively. This construction reflects a spatial organization that cells further away spatially have higher probability of being extruded. In order to maintain a static equilibrium, the sizes of the first three demes were chosen to be 20, 45 and 85 respectively.

**C)** Continuous time models. In the continuous time model, each cell is dividing at a rate λ. When there are n cells, the time to the next event is exponentially distributed with rate nλ. Given the time to the next event, the exact cell which experiences this event will be randomly picked among n cells following statistical properties of the Poisson Process. In our setting, there will be N_1_ stem cells and N_2_ transit amplifying cells. When a stem cell is picked, it will be chosen to have type I/II/III divisions with the associated probabilities specified by the α and β parameters. When a transit amplifying cell is picked, it can either divide (with probability γ) or being extruded out of the crypt (with probability 1-γ). In our setting, we choose γ to be (N_2_-N_1_)/2N_2_. In order to maintain a static range of population sizes, we used rejection sampling to constraint the stem cell population size to be between 10 and 20. This is realized by rejecting certain moves in the process when the proposed move is taking the population out of this range .

Given the sampled genealogy at the end of the simulation run, the exact cell division number is also recorded in the associated tree structure. The subsequent calculation will be the similar to other models.

**Part three: Crypt history and gene genealogies at day 52**

Following previous experimental evidences as well as predictions from the optimal control theory , crypt history follows two phases, namely morphogenesis and homeostasis (Figure 3). Intestinal crypt is formed by first creating N1 stem cells starting from a single stem cell through symmetric stem cell divisions. After creating N1 stem cells, stem cells will follow strictly asymmetric divisions until crypt formation. Meanwhile, non-stem cells also divide and give rise to non-stem descendants. Crypt morphogenesis ends when there are N2 non-stem cells in the intestinal crypts. After crypt morphogenesis, populations follow the dynamic process described in the two-deme population genetic model (Materials and Methods). At the end of our simulations, a random subset of cells is sampled and their genealogical history is recorded.

Since we know that crypt morphogenesis starts around post-natal day 7 for mice and stem cells divide every 22 hours , postnatal day 52 corresponds to about generation 42 after crypt formation. Considering the fact that there might be random variation around this mean value, we capture this level of variability by using various random distributions. For example, we used a beta distribution y=40×beta(p, q) +35 for cell generation number (p=3 and q=5 for beta distribution) for the results shown in the main text. In addition, we also tried truncated normal distributions with different density shapes for the cell generation numbers and the results stay quantitatively very similar (data not shown).

**Part four: mutation rates and the likelihood calculation**

In order to take into account variability in the mutation rates, we used various forms of beta distibutions to capture this variability. Since the mutation rate measured from previous experimental studies is 0.01 per site per generation. We used beta distributions ranging between 0.0075 and 0.0125 with different shape parameters to capture possible variation in mutation rates. In particular, we used beta (p,q) where p and q are the two parameters of the beta distribution. We set p to be equal to q such that the distribution is symmetric around the mean value. Higher p will lead to shaper distributions (less variability) around the mean value. We tried p with three different values (2,3,5).

The likelihood of the data can be calculated by partitioning the mutation distribution into discrete bins and take the weighted sum of individual likelihoods calculated at discrete points of mutation rates . We find that the likelihood function gives similar point estimates for the different distributions of the mutation rates (Figure S3).

**References**

1. Potten CS, Loeffler M (1990) Stem cells: attributes, cycles, spirals, pitfalls and uncertainties. Lessons for and from the crypt. Development 110: 1001-1020.

2. Ross MS (2006) Simulation: Academic Press. 312 p.

3. Itzkovitz S, Blat IC, Jacks T, Clevers H, van Oudenaarden A (2012) Optimality in the development of intestinal crypts. Cell 148: 608-619.

4. Cheng H, Bjerknes M (1985) Whole population cell kinetics and postnatal development of the mouse intestinal epithelium. Anat Rec 211: 420-426.

5. Dehmer JJ, Garrison AP, Speck KE, Dekaney CM, Van Landeghem L, et al. (2011) Expansion of intestinal epithelial stem cells during murine development. PLoS One 6: e27070.

6. Snippert HJ, van der Flier LG, Sato T, van Es JH, van den Born M, et al. (2010) Intestinal crypt homeostasis results from neutral competition between symmetrically dividing Lgr5 stem cells. Cell 143: 134-144.

7. Yang Z (1994) Maximum likelihood phylogenetic estimation from DNA sequences with variable rates over sites: approximate methods. J Mol Evol 39: 306-314.
